# Supplementary material for: PBPK Modelling of Dexamethasone in Patients With COVID-19 and Liver Disease
Source: Front Pharmacol. 2022 Jan 28;13:814134. doi: 10.3389/fphar.2022.814134 (PMC8832977; doi:10.3389/fphar.2022.814134)
Supplement: Supplementary file 1 [file DataSheet1.DOCX]

**PBPK modelling of dexamethasone in patients with COVID-19 and liver disease**

Maiara Camotti Montanha, Ph.D.^1^, Nicolas Cottura, PharmD^1^, Michael Booth, MSc.^1^, Daryl Hodge, Ph.D.^1^, Fazila Bunglawala, MSc.^1^, Hannah Kinvig, Ph.D.^1^, Sandra Grañana-Castillo^1^, Andrew Lloyd^1^, Saye Khoo, M.D.^1^, Marco Siccardi, Ph.D.^1^.

^1^Department of Pharmacology and Therapeutics, Institute of Systems, Molecular and Integrative Biology, University of Liverpool, Liverpool, United Kingdom

Author for correspondence: Dr. Maiara Montanha, Department of Pharmacology and Therapeutics, Institute of Systems, Molecular and Integrative Biology, University of Liverpool, 70 Pembroke Place, Liverpool, L69 3GF, U.K.

Tel No +44 (0) 151 794 8211

Fax No + 44 (0) 151 794 5656

E-mail: M.camotti-montanha@liverpool.ac.uk

**Supplementary material**

**Figure S1.** Simulated *versus* observed ^1^ mean plasma concentration of midazolam after administration of 7.5 mg intravenously (IV) and 15 mg orally (PO) in a healthy population.

**Figure S2.** Simulated *versus* observed ^1^ mean plasma concentration of midazolam after administration of 7.5 mg intravenously (IV) and 15 mg orally (PO) considering the mean of Child-Pugh (CP)-A, CP-B and CP-C liver disease classification.

**Figure S3.** Simulated *versus* observed ^2^ mean plasma concentration of propranolol after administration of 1 mg intravenously (IV) and 40 mg orally (PO) in a healthy population.

**Figure S4.** Simulated *versus* observed ^2^ mean plasma concentration of propranolol after administration of 1 mg intravenously (IV) and 40 mg orally (PO) considering the mean of Child-Pugh (CP)-A, CP-B and CP-C liver disease classification.

**Figure S5.** Simulated *versus* observed ^3^ mean plasma concentration of dexamethasone after administration of 5 mg intravenously (IV) and 4.5 mg orally (PO) in a healthy population.

**Table S1.** Mean simulated PK parameters of midazolam (MDZ) in different liver disease conditions.

|  | MDZ 7.5 mg IV | | | | | | MDZ 15 mg PO | | | | | |
| --- | --- | --- | --- | --- | --- | --- | --- | --- | --- | --- | --- | --- |
|  | AUC_0-inf_ | AFE | CL | AFE | t_1/2_ | AFE | AUC_0-inf_ | AFE | C_max_ | AFE | t_max_ | AFE |
| Simulated CP-A | 397.67 | 1.37 | 251.47 | 1.25 | 3.97 | 1.85 | 389.02 | 1.48 | 69.18 | 1.40 | 0.75 | 1.00 |
| Simulated CP-B | 472.48 | 1.15 | 211.65 | 1.06 | 4.94 | 1.49 | 426.66 | 1.35 | 65.40 | 1.48 | 0.75 | 1.00 |
| Simulated CP-C | 535.03 | 1.01 | 186.91 | 1.07 | 6.04 | 1.22 | 538.19 | 1.07 | 75.07 | 1.29 | 0.75 | 1.00 |
| Simulated mean CP | 468.39 | 1.16 | 213.50 | 1.07 | 5.06 | 1.45 | 451.29 | 1.28 | 69.88 | 1.39 | 0.75 | 1.19 |

Data are presented as the mean. AFE, absolute average-fold error calculated considering observed data [1]. AUC_0-inf_, area under the plasma concentration-time curve over a dosing interval (ng·h/mL). C_max_ maximum plasma concentration (ng/mL). t_1/2_, half-life time (h). CL, clearance (mL/h/kg). t_max_, time to maximum plasma concentration (h).

**Table S2.** Individual simulated pharmacokinetic parameters of propranolol (PRO) matching individual age, Child-Pugh score, and shunt index of observed data.^2^

|  |  |  |  |  | PRO 1 mg IV | | | | | | PRO 40 mg PO | | | | | |
| --- | --- | --- | --- | --- | --- | --- | --- | --- | --- | --- | --- | --- | --- | --- | --- | --- |
| ID | Age | BMI | CP | Shunt  Index (%) | AUC_0-inf_ | | | CL | | | AUC_0-inf_ | | | C_max_ | | |
|  |  |  |  |  | Simulated | Clinical | AFE | Simulated | Clinical | AFE | Simulated | Clinical | AFE | Simulated | Clinical | AFE |
| 1 | 57 | 20.4 | B | 7 | 1832 | 889 | 2.06 | 734 | 1125 | 1.53 | 26149 | 39289 | 1.50 | 48 | 90 | 1.89 |
| 2 | 51 | 31.2 | A | 21 | 1240 | 1818 | 1.47 | 1080 | 550 | 1.96 | 20319 | 48787 | 2.40 | 50 | 59 | 1.19 |
| 5 | 47 | 21.2 | A | 12 | 1221 | 883 | 1.38 | 1108 | 1133 | 1.02 | 16339 | 22707 | 1.39 | 40 | 25 | 1.60 |
| 6 | 37 | 24.2 | A | 9 | 1217 | 791 | 1.54 | 1109 | 1265 | 1.14 | 14984 | 14143 | 1.06 | 36 | 50 | 1.39 |
| 7 | 50 | 25.4 | C | 64 | 2383 | 1752 | 1.36 | 559 | 571 | 1.02 | 71461 | 72859 | 1.02 | 109 | 63 | 1.73 |
| 8^*^ | 49 | 28.1 | B | 59 | 1792 | 7200 | 4.02 | 750 | 139 | 5.40 | 46360 | 88124 | 1.90 | 90 | 62 | 1.46 |
| 9 | 50 | 29.6 | C | 31 | 2475 | 1264 | 1.96 | 533 | 791 | 1.48 | 55098 | 49476 | 1.11 | 81 | 33 | 2.46 |
| 10 | 60 | 31.8 | A | 16 | 1330 | 1432 | 1.08 | 1020 | 698 | 1.46 | 20667 | 17894 | 1.15 | 48 | 20 | 2.41 |
| 11^*^ | 60 | 30.4 | A | 24 | 1302 | 1536 | 1.18 | 1033 | 651 | 1.59 | 21844 | 76244 | 3.49 | 53 | 132 | 2.48 |
| 12 | 51 | 20.4 | B | 43 | 1775 | 1964 | 1.11 | 754 | 606 | 1.24 | 40992 | 66527 | 1.62 | 80 | 32 | 2.51 |
| 13 | 58 | 21.3 | B | 22 | 1931 | 2540 | 1.32 | 699 | 394 | 1.77 | 35106 | 42261 | 1.20 | 63 | 53 | 1.18 |
| 14 | 67 | 26.7 | A | 13 | 1356 | 1004 | 1.35 | 1001 | 996 | 1.00 | 20577 | 44270 | 2.15 | 48 | 82 | 1.71 |
| 15^*^ | 50 | 23.5 | B | 19 | 1778 | 505 | 3.52 | 756 | 1981 | 2.62 | 30022 | 23729 | 1.27 | 56 | 59 | 1.05 |
| All |  |  |  |  | 1676 | 1778 | 1.06 | 597 | 833 | 1.40 | 34258 | 47260 | 1.38 | 60 | 65 | 1.08 |

Data are presented as the mean. ^*^outliers individuals. AFE, absolute average-fold error calculated considering observed data. Age, years. CP, Child-Pugh score. AUC_0-inf_, area under the plasma concentration-time curve over a dosing interval (ng.min/mL). C_max_ maximum plasma concentration (ng/mL). CL, clearance (mL/min).

**Table S3.** Mean simulated pharmacokinetic parameters of dexamethasone (DEX) in different liver disease conditions.

|  |  | IV | | | PO | | | | *f*_up_ |
| --- | --- | --- | --- | --- | --- | --- | --- | --- | --- |
| Dose | Population | AUC_0-24_ | CL | Vd | AUC_0-24_ | C_max_ | CL | Vd |  |
| 6 mg | Healthy | 366.61 (18) | 16.36 (14) | 87.21 (10) | 320.64 (21) | 48.30 (17) | 18.70 (13) | 100.31(13) | 0.23 |
|  | CP-A | 533.03 (17) | 11.25 (12) | 78.03 (13) | 484.15 (22) | 61.44 (19) | 12.38 (16) | 85.21 (11) | 0.25 |
|  | CP-B | 652.73 (17) | 9.18 (14) | 74.95 (12) | 590.51 (18) | 66.81 (16) | 10.15 (12) | 81.81 (11) | 0.28 |
|  | CP-C | 782.46 (14) | 7.66 (12) | 73.54 (12) | 708.24 (16) | 69.20 (15) | 8.46 (12) | 82.94 (11) | 0.34 |
|  | CP-A_shunt_ | 525.04 (17) | 11.42 (14) | 78.23 (11) | 512.23 (17) | 62.22 (15) | 11.70 (12) | 83.86 (11) | 0.25 |
|  | CP-B_shunt_ | 646.17 (16) | 9.28 (12) | 76.76 (11) | 620.46 (18) | 68.29 (16) | 9.66 (12) | 78.79 (12) | 0.28 |
|  | CP-C_shunt_ | 785.94 (17) | 7.63 (12) | 71.48 (12) | 749.54 (18) | 75.73 (18) | 8.00 (12) | 75.21 (12) | 0.34 |
| 20 mg | Healthy | 1190.15 (17) | 16.80 (12) | 90.15 (13) | 1051.33 (17) | 158.44 (15) | 19.02 (12) | 102.14 (12) | 0.23 |
|  | CP-A | 1748.29 (19) | 11.43 (14) | 78.82 (10) | 1616.10 (18) | 202.82 (17) | 12.37 (12) | 85.67 (11) | 0.25 |
|  | CP-B | 2118.08 (16) | 9.43 (12) | 77.09 (12) | 1944.64 (17) | 216.31 (14) | 10.28 (12) | 83.72 (12) | 0.28 |
|  | CP-C | 2547.98 (15) | 7.84 (12) | 72.20 (12) | 2269.89 (19) | 230.72 (15) | 8.80 (12) | 82.44 (11) | 0.34 |

Data are presented as the mean (coefficient of variation, %). AUC_0-24_, area under the plasma concentration-time curve over a dosing interval (ng.h/mL). C_max_ maximum plasma concentration (ng/mL). CL, clearance (L/h). Vd, volume of distribution (L). *f*_up_, fraction unbound in plasma. CP-A, CP-B, CP-C and CP-A_shunt_, CP-B_shunt_, CP-C_shunt_ correspond to the Child-Pugh score without and with shunting incorporated, respectively. IV and PO, intravenous and oral administration.

**References**

1. Pentikäinen PJ, Välisalmi L, Himberg JJ, Crevoisier C. Pharmacokinetics of midazolam following intravenous and oral administration in patients with chronic liver disease and in healthy subjects. *J Clin Pharmacol.* 1989;29(3):272-277.

2. Taegtmeyer AB, Haschke M, Tchambaz L, et al. A study of the relationship between serum bile acids and propranolol pharmacokinetics and pharmacodynamics in patients with liver cirrhosis and in healthy controls. *PLoS One.* 2014;9(6):e97885.

3. Varis T, Kivistö KT, Backman JT, Neuvonen PJ. The cytochrome P450 3A4 inhibitor itraconazole markedly increases the plasma concentrations of dexamethasone and enhances its adrenal-suppressant effect. *Clin Pharmacol Ther.* 2000;68(5):487-494.
